# Propensity score matching comparisons of postoperative complications and morbidity between digestive tract reconstruction methods after gastrectomy in gastric cancer patients with visceral obesity

**DOI:** 10.3389/fonc.2022.1072127

**Published:** 2023-02-13

**Authors:** Chenchen Mao, Miaofang Xiao, Jian Chen, Jian Wen, Hui Yang, Wentao Cai, Jingwei Zheng, Xinxin Chen, Xiaofeng Xing, Xiangyang Xue, Xian Shen, Sini Wang

**Affiliations:** ^1^ Department of Gastrointestinal Surgery, The First Affiliated Hospital, Wenzhou Medical University, Wenzhou, Zhejiang, China; ^2^ Department of Medical Microbiology and Immunology, Basic Medical College, Wenzhou Medical University, Wenzhou, Zhejiang, China; ^3^ Department of Gastrointestinal Surgery, The Second Affiliated Hospital, Wenzhou Medical University, Wenzhou, Zhejiang, China; ^4^ Wenzhou Collaborative Innovation Center of Gastrointestinal Cancer in Basic Research and Precision Medicine, Wenzhou Key Laboratory of Cancer-related Pathogens and Immunity, Experiential Center of Basic Medicine, Department of Microbiology and Immunology, Institute of Molecular Virology and Immunology, School of Basic Medical Sciences, Wenzhou Medical University, Wenzhou, Zhejiang, China; ^5^ Department of Radiology, The First Affiliated Hospital, Wenzhou Medical University, Wenzhou, Zhejiang, China

**Keywords:** abdominal obesity, stomach neoplasms, reconstruction, postoperative complications, propensity score matching

## Abstract

**Background:**

Few studies have compared the prognosis of different reconstruction methods after gastrectomy for gastric cancer (GC) patients with obesity. The aim of the present study was to compare postoperative complications and overall survival (OS) between the following reconstruction methods: Billroth I (B-I), Billroth II (B-II), and Roux-en-Y (R-Y) after gastrectomy for GC patients with visceral obesity (VO).

**Methods:**

We performed a double-institutional dataset study of 578 patients who underwent radical gastrectomy with B-I, B-II, and R-Y reconstructions between 2014 and 2016. VO was defined as a visceral fat area at the level of the umbilicus greater than 100 cm^2^. Propensity score-matching analysis was performed to balance the significant variables. Postoperative complications and OS were compared between the techniques.

**Results:**

VO was determined in 245 patients, of which 95, 36, and 114 underwent B-I, B-II, and R-Y reconstructions, respectively. B-II and R-Y were fused into the Non-B-I group due to the similar incidence of overall postoperative complications and OS. Therefore, 108 patients were enrolled after matching. The overall postoperative complications incidence and overall operative time in the B-I group were significantly lower than those in the non-B-I group. Further, multivariable analysis showed that B-I reconstruction was an independent protective factor for overall postoperative complications (odds ratio (OR) 0.366, P=0.017). However, no statistical difference in OS was found between the two groups (hazard ratio (HR) 0.644, P=0.216).

**Conclusions:**

B-I reconstruction was associated with decreased overall postoperative complications, rather than OS, in GC patients with VO who underwent gastrectomy.

## Introduction

Gastric cancer (GC) is one of the most common cancer types and the leading cause of cancer-related mortality worldwide (1). Although various treatments such as chemotherapy, chemoradiotherapy, targeted therapy, and immunotherapy have been developed (2, 3), radical gastrectomy remains the most effective. Reconstruction methods such as Billroth I (B-I), Billroth II (B-II), and Roux-en-Y (R-Y) are commonly used after gastrectomy (4, 5). The choice of the reconstruction method is mostly based on the patient’s condition and the surgeon’s preference. Previous studies have compared B-I and R-Y reconstruction methods with inconsistent results; while some of the studies demonstrated that B-I reconstruction was preferable with decreasing overall postoperative complications (6) and morbidity (7), another one showed no significant differences between the two groups in the long-term patients’ quality of life and the incidence of the postoperative complications (8, 9). Moreover, an additional study reported that R-Y reconstruction does not have greater postoperative complications than B-II does (10). Thus, the selection of the most appropriate reconstruction method after gastrectomy remains controversial.

Obesity, whether determined by body mass index (BMI) or visceral fat area measurements, has been reported to be associated with a higher incidence of postoperative complications for GC (11, 12). Indeed, the narrow operation space and exposure difficulty attributed to the abdominal wall’s obesity-related hypertrophy and the greater omentum increase the difficulty of specimen isolation and digestive tract reconstruction. Our previous study (13) revealed that laparoscopic gastrectomy significantly decreased the rate of these postoperative complications of GC patients with visceral obesity (VO) owing to the advantages concerning the visual field and operating space. Similarly, different methods of digestive tract reconstruction have different requirements related to the degree of tissue dissociation, leading to differences in operation time and possible tissue injury resulting from the traction applied during the operation, which may also greatly affect the incidence of intraoperative and postoperative complications, especially in VO patients (14). Despite all this, few studies focused on the prognosis of GC after digestive tract reconstruction.

In this study, we used propensity-score-matching (PSM) to balance the significant variables strictly and further compared the incidence of postoperative complications and survival upon different digestive tract reconstructions after gastrectomy in GC patients with obesity. In this way, we aim to obtain clinical evidence for selecting the most appropriate digestive tract reconstruction after gastrectomy in VO patients.

## Materials and methods

### Study design and patient population

**Figure 1:**
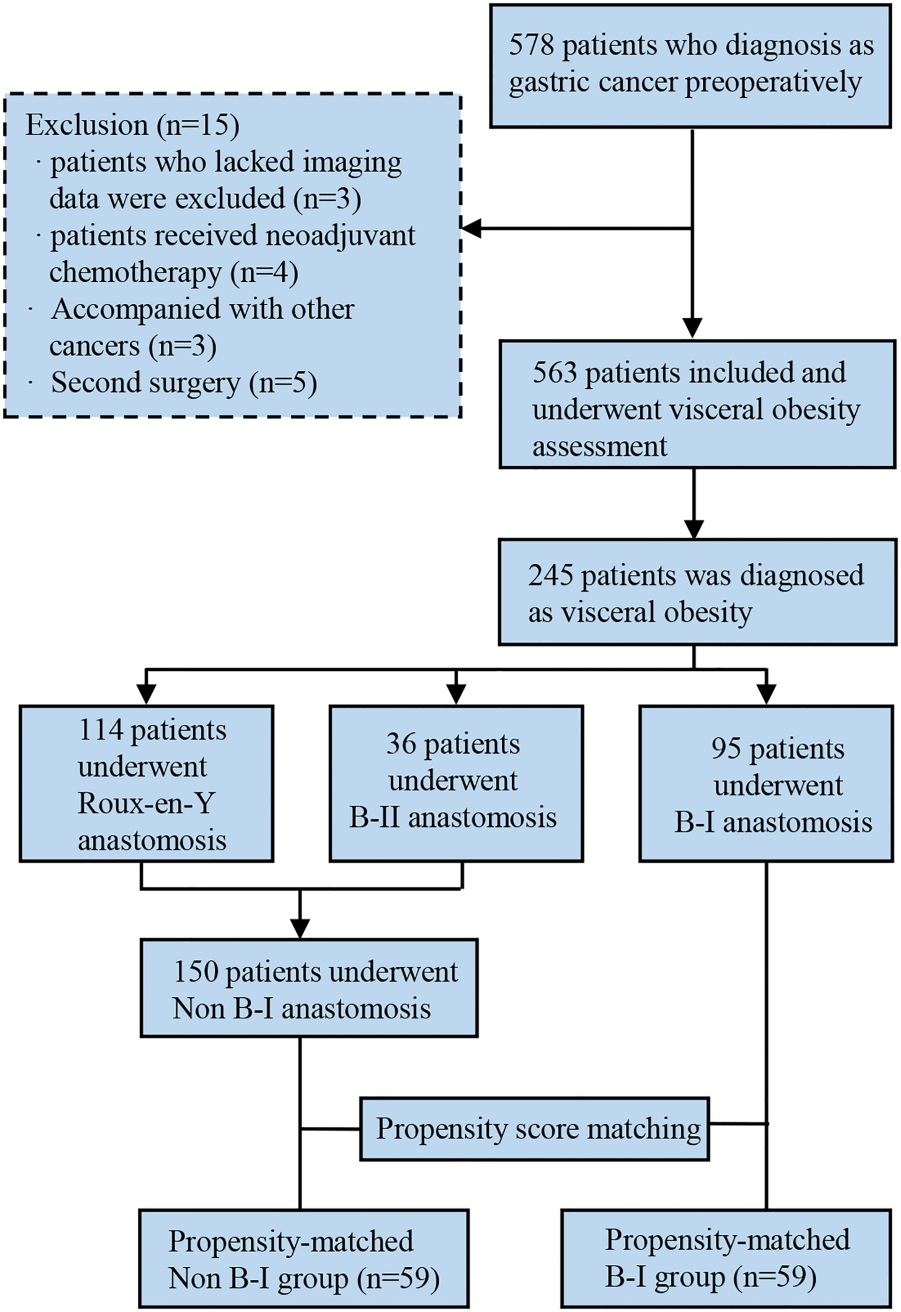
Flow diagram of the study process.

Clinical data of 578 GC patients who underwent curative gastrectomy and D2 lymph node dissection at the Gastrointestinal Surgical Departments of the Second Affiliated Hospital of Wenzhou Medical University and the First Affiliated Hospital of Wenzhou Medical University in China were retrospectively collected between January 2014 and December 2016. Patients were enrolled for analysis based on the following criteria: (1) underwent gastrectomy and confirmed as gastric adenocarcinoma by postoperative pathology; (2) older than 18 years. The exclusion criteria were as follows: (1) lack of imaging data; (2) lack of clinical data; (3) underwent palliative or emergency surgery; (4) received preoperative neoadjuvant chemotherapy or radiotherapy; (5) accompanied with severe immune, blood, or endocrine disease; (6) GC concurrent with other malignant tumors. The outline of this study is summarized in **Figure 1**.

### Baseline data collection

For each patient enrolled in this study, demographic details, including age, sex, BMI, American Society of Anesthesiologists (ASA) grade, abdominal operation history, and NRS 2002 score, were collected, along with details on the operation, such as tumor location, tumor differentiation, pathological classification, and histopathologic staging according to TNM staging (AJCC Cancer Staging System, 8^th^ ed). Additionally, postoperative complications were defined as adverse events occurring within 90 days after surgery, according to the Clavien–Dindo classification system (15). Patients with more than two complications were classified as having multiple complications. Postoperative hospital stay, hospitalization costs, and OS were also recorded.

### Computed tomography-based measurement of visceral fat area

Preoperatively, all patients underwent computed tomography (CT) of the general abdominal cavity. A single scan in a cross-section at the level of the umbilicus was selected to quantify the degree of visceral fat. Visceral fat was measured under a threshold of -140 to -50 as reported in the previous studies (16, 17). The total fat area was calculated using a dedicated processing system (version 3.0.11.3, BN17 32-bit; INFINITT Healthcare Co. Ltd., Seoul, South Korea). VO was determined as having a visceral fat area (VFA) of more than 100 cm^2^ (18, 19).

### PSM and statistical analyses

PSM was performed to balance the significant variables in the following analyses strictly. Propensity scores were generated using a logistic regression model on covariates with differences before matching: age, tumor location, TNM stage, combined organ resection, previous abdominal surgery and laparoscopic gastrectomy. PSM was performed in a 1:1 ratio using a 0.03 caliper width, and the resulting score-matched pairs were used in subsequent analyses. The two matched groups were evaluated for the study endpoints. Means and standard deviations were used for all continuous data, and numbers and percentages were calculated for all categorical data. Intergroup differences in clinicopathological variables were analyzed using the chi-square test or Fisher’s exact test for categorical data and the Mann–Whitney U test for continuous data. We also performed conditional logistic regression analyses after the relevant prognostic variables were defined using univariate analysis. Overall survival (OS) was determined as the time between the diagnosis and death or the last follow-up date. Kaplan–Meier and log-rank tests were performed to estimate and compare survival rates, respectively. The Cox proportional hazard model was performed to estimate the risk ratio in the univariate and multivariate analyses, and the results were expressed as hazard ratios (HRs) with 95% confidence intervals (CIs). Statistical significance was set at P < 0.05. All statistical analyses were performed using SPSS version 22.0 (SPSS Inc., Chicago, IL, USA) and R version 3.0.1 (http://www.Rproject.org).

## Results

### Patient characteristics

We included 245 patients with VO and GC in this study. A total of 95 patients (38.78%) underwent B-I reconstruction, 36 patients (14.69%) underwent B-II reconstruction, and 114 patients (46.53%) underwent R-Y reconstruction.

**Figure 2:**
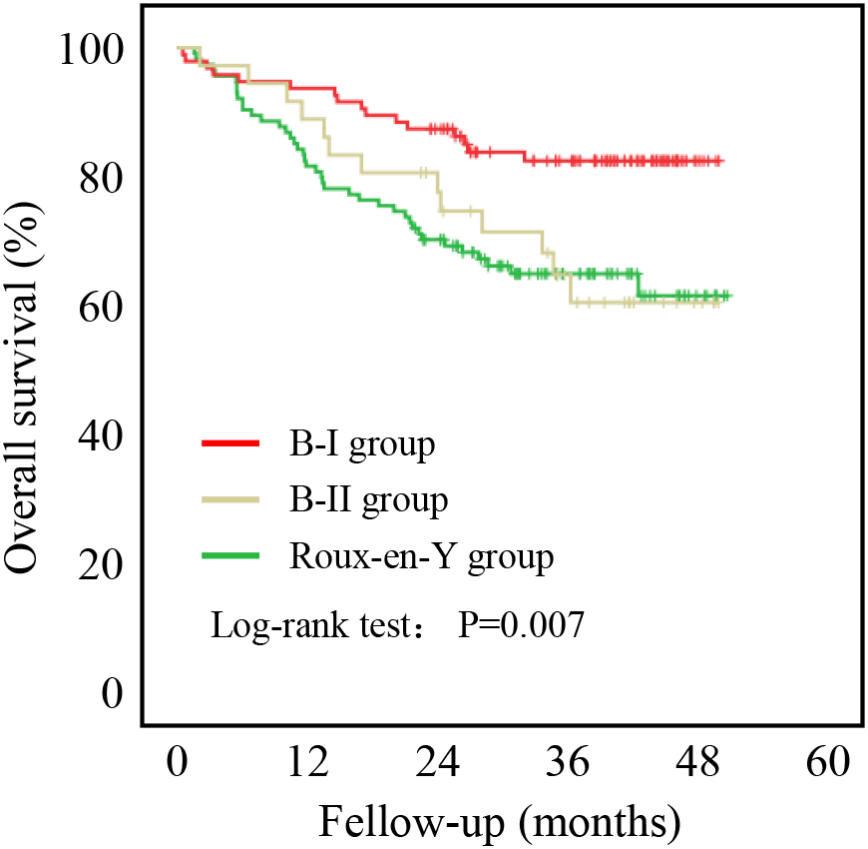
Five-year overall survival curve calculated using the Kaplan-Meier method comparing B-I, B-II, and R-Y reconstructions.

Patients who underwent B-I reconstruction showed a better prognosis outcome than those in the other two groups, as well as the lowest rate of postoperative complications (**Table 1**) and best OS (**Figure 2**). However, as summarized in **Table 1**, B-I reconstruction was more likely to be performed in patients who underwent laparoscopic gastrectomy (P=0.002) and primary focus resection only (P=0.002). Furthermore, patients who underwent B-I reconstruction were classified as having a lower TNM stage (P<0.001). Considering the limited sample size and differences in clinical characteristics, we fused the B-II and R-Y groups into the non-B-I group. PSM was further performed to minimize selection bias, resulting in the clinicopathological characteristics of the Non-B-I and B-I groups (n = 59 for each group) being well balanced (**Table 2**).

**Table 1:** Patient baseline characteristics.

Factors	B-I (n=95)	B-II (n=36)	R-Y (n=114)	P
Gender				0.269
Male	76 (80.00%)	24 (66.67%)	88 (77.19%)	
Female	19 (20.00%)	12 (33.33%)	26 (22.81%)	
Age (y)				0.066
≤65	51 (53.68%)	13 (36.11%)	45 (39.47%)	
>65	44 (46.32%)	23 (63.89%)	69 (60.53%)	
NRS 2002 score				0.243
1-2	72 (75.79%)	22 (61.11%)	71 (62.28%)	
3-4	19 (20.00%)	11 (30.56%)	33 (28.95%)	
5-6	4 (4.21%)	3 (8.33%)	10 (8.77%)	
ASA grade				0.096
1-2	76 (80.00%)	24 (66.67%)	95 (83.33%)	
3-4	19 (20.00%)	12 (33.33%)	19 (16.67%)	
Hypertension				0.277
Yes	29 (30.53%)	13 (36.11%)	47 (41.23%)	
No	66 (69.47%)	23 (63.89%)	67 (58.77%)	
Diabetes mellitus				0.195
Yes	14 (14.74%)	10 (27.78%)	25 (21.93%)	
No	81 (85.26%)	26 (72.22%)	89 (78.07%)	
Previous abdominal surgery				0.395
Yes	10 (10.53%)	7 (19.44%)	13 (11.40%)	
No	85 (89.47%)	29 (80.56%)	10 (8.77%)	
Tumor location				
Cardia	0 (0.00%)	0 (0.00%)	38 (33.33%)	<0.001*
body	83 (87.37%)	33 (91.67%)	35 (30.70%)	
Antrum	10 (10.53%)	3 (8.33%)	34 (29.82%)	
Total	1 (1.05%)	0 (0.00%)	7 (6.14%)	
Differentiated degree				0.347
Differentiated	69 (72.63%)	29 (80.56%)	88 (77.19%)	
Undifferentiated	8 (8.42%)	2 (5.56%)	14 (12.28%)	
Signet ring carcinoma	18 (18.95%)	5 (13.89%)	12 (10.53%)	
Pathological type				0.085
Ulcerative type	83 (87.37%)	35 (97.22%)	97 (85.09%)	
Non-ulcerative type	12 (12.63%)	1 (2.78%)	17 (14.91%)	
TNM stage				<0.001*
I	50 (52.63%)	7 (19.44%)	25 (21.93%)	
II	20 (21.05%)	11 (30.56%)	32 (28.07%)	
III	25 (26.32%)	18 (50.00%)	57 (50.00%)	
Combined organ resection				0.002*
Yes	1 (1.05%)	2 (5.56%)	14 (12.28%)	
No	94 (98.95%)	34 (94.44%)	10 (8.77%)	
Laparoscopic gastrectomy				0.002*
Yes	33 (34.74%)	3 (8.33%)	21 (18.42%)	
No	62 (65.26%)	33 (91.67%)	93 (81.58%)	
Postoperative complications				0.026*
Yes	23 (24.21%)	18 (50.00%)	48 (42.11%)	
No	72 (75.79%)	18 (50.00%)	66 (57.89%)	

BMI body mass index, ASA American Society of Anesthesiologists, NRS 2002 nutritional risk screening 2002, TNM tumor-node-metastasis.

The values given are number of patients and values in parentheses are percentages.

*Represent P < 0.05, which was considered to be statistically significant.

**Table 2:** Patient baseline characteristics.

Factors	Unmatched			Matched		
	B-I (n=95)	Non-B-I (n=150)	P	B-I (n=59)	Non-B-I (n=59)	P
Gender			0.336			0.285
Male	76 (80.00%)	112 (74.67%)		47 (79.66%)	42 (71.19%)	
Female	19 (20.00%)	38 (25.33%)		12 (20.34%)	17 (28.81%)	
Age (y)			0.021*			1.000
≤65	51 (53.68%)	58 (38.67%)		25 (42.37%)	25 (42.37%)	
>65	44 (46.32%)	92 (61.33%)		34 (57.63%)	34 (57.63%)	
NRS 2002 score			0.071			0.567
1-2	72 (75.79%)	93 (62.00%)		41 (69.49%)	38 (64.41%)	
3-4	19 (20.00%)	44 (29.33%)		15 (25.42%)	15 (25.42%)	
5-6	4 (4.21%)	13 (8.67%)		3 (5.08%)	6 (10.17%)	
ASA grade			0.900			0.831
1-2	76 (80.00%)	119 (79.33%)		45 (76.27%)	44 (74.58%)	
3-4	19 (20.00%)	31 (20.67%)		14 (23.73%)	15 (25.42%)	
Hypertension			0.133			0.334
Yes	29 (30.53%)	60 (40.00%)		23 (38.98%)	18 (30.51%)	
No	66 (69.47%)	90 (60.00%)		36 (61.02%)	41 (69.49%)	
Diabetes mellitus			0.101			0.499
Yes	14 (14.74%)	35 (23.33%)		11 (18.64%)	14 (23.73%)	
No	81 (85.26%)	115 (76.67%)		48 (81.36%)	45 (76.27%)	
Previous abdominal surgery			0.515			0.071
Yes	10 (10.53%)	20 (13.33%)		5 (8.47%)	13 (22.03%)	
No	85 (89.47%)	130 (86.67%)		54 (91.53%)	46 (77.97%)	
Tumor location			<0.001*			1.000
Cardia	0 (0.00%)	38 (25.33%)		0 (0.00%)	2 (3.39%)	
body	83 (87.37%)	68 (45.33%)		50 (84.75%)	48 (81.36%)	
Antrum	10 (10.53%)	37 (24.67%)		9 (15.25%)	7 (11.86%)	
Total	1 (1.05%)	7 (4.67%)		0 (0.00%)	2 (3.39%)	
Differentiated degree			0.237			0.972
Differentiated	69 (72.63%)	117 (78.00%)		41 (69.49%)	40 (67.8%)	
Undifferentiated	8 (8.42%)	16 (10.67%)		7 (11.86%)	7 (11.86%)	
Signet ring carcinoma	18 (18.95%)	17 (11.33%)		11 (18.64%)	12 (20.34%)	
Pathological type			0.883			0.113
Ulcerative type	83 (87.37%)	132 (88.00%)		51 (86.44%)	56 (94.92%)	
Non-ulcerative type	12 (12.63%)	18 (12.00%)		8 (13.56%)	3 (5.08%)	
TNM stage			<0.001*			0.833
I	50 (52.63%)	32 (21.33%)		19 (32.2%)	16 (27.12%)	
II	20 (21.05%)	43 (28.67%)		15 (25.42%)	16 (27.12%)	
III	25 (26.32%)	75 (50.00%)		25 (42.37%)	27 (45.76%)	
Combined organ resection			0.004*			1.000
Yes	1 (1.05%)	16 (10.67%)		1 (1.69%)	1 (1.69%)	
No	94 (98.95%)	134 (89.33%)		58 (98.31%)	58 (98.31%)	
Laparoscopic gastrectomy			0.001*			1.000
Yes	33 (34.74%)	24 (16.00%)		13 (22.03%)	13 (22.03%)	
No	62 (65.26%)	126 (84.00%)		46 (77.97%)	46 (77.97%)	

BMI body mass index, ASA American Society of Anesthesiologists, NRS 2002 nutritional risk screening 2002, TNM tumor-node-metastasis.

The values given are number of patients and values in parentheses are percentages.-

*Represent P < 0.05, which was considered to be statistically significant.

### Surgical outcomes and postoperative course

Further analyses were performed using data from 118 patients after PSM. The operation time for the B-I group (193.88 ± 48.16 min) was significantly shorter than for the Non-B-I group (212.95 ± 48.54 min, P = 0.034) and the postoperative hospital stay was shorter (15.22 ± 7.13 days VS 18.29 ± 9.58 days, P = 0.050) in the B-I group. As for the hospitalization costs, no significant differences were found between the two groups (68142.02 ± 26214.52 Yuan vs. 66320.71 ± 17834.16 Yuan, P = 0.660). The overall incidence of postoperative complications was significantly lower in the B-I group (25.42%, 15/59) than in the non-B-I group (45.76%, 27/59) (P = 0.021). Further analyses showed that both surgical and medical complications incidence tended to be lower in the B-I group, although the difference was not statistically significant. (**Table 3**).

**Table 3:** Surgical outcomes before and after matching.

Factors	Unmatched				Matched			
	Total (n=245)	B-I (n=95)	Non-B-I (n=150)	P	Total (n=118)	B-I (n=59)	Non-B-I (n=59)	P
Operative time, (X ± SD), min	206.84 ± 50.23	190.13 ± 48.01	217.30 ± 48.92	<0.001*	203.42 ± 49.09	193.88 ± 48.16	212.95 ± 48.54	0.034*
Postoperative hospital stays, (X ± SD), days	15.85 ± 8.62	14.30 ± 7.65	16.83 ± 9.07	0.020*	16.75 ± 8.55	15.22 ± 7.13	18.29 ± 9.58	0.050*
Hospitalization costs, (X ± SD), yuan	65915.22 ± 32021.88	63459.53 ± 26949.49	67470.49 ± 34852.73	0.313	67231.36 ± 22342.10	68142.02 ± 26214.52	66320.71 ± 17834.16	0.660
Total complications^a^	89 (36.32%)	23 (24.21%)	66 (44.00%)	0.002*	42 (35.59%)	15 (25.42%)	27 (45.76%)	0.021*
Clavien-Dindo grade								
Grade I	8 (3.27%)	0 (0.00%)	8 (5.33%)	0.025*	5 (4.24%)	0 (0.00%)	5 (8.47%)	0.068
Grade II	58 (23.67%)	16 (16.84%)	42 (28.00%)	0.045*	26 (22.03%)	10 (16.95%)	16 (27.12%)	0.183
Grade III	15 (6.12%)	4 (4.21%)	11 (7.33%)	0.321	7 (5.93%)	3 (5.08%)	4 (6.78%)	1.000
Grade IV	8 (3.27%)	3 (3.16%)	5 (3.33%)	1.000	4 (3.39%)	2 (3.39%)	2 (3.39%)	1.000
Detail of complications								
Surgical complications	40 (16.33%)	10 (10.53%)	30 (20.00%)	0.051	18 (15.25%)	7 (11.86%)	11 (18.64%)	0.306
Gastrointestinal dysfunction	7 (2.86%)	0 (0.00%)	7 (4.67%)	0.081	4 (3.39%)	0 (0.00%)	4 (6.78%)	0.127
Intestinal obstruction	3 (1.22%)	1 (1.05%)	2 (1.33%)	1.000	3 (2.54%)	1 (1.69%)	2 (3.39%)	1.000
Anastomotic leakage	2 (0.82%)	0 (0.00%)	2 (1.33%)	0.523	1 (0.85%)	0 (0.00%)	1 (1.69%)	1.000
Severe wound infection	5 (2.04%)	1 (1.05%)	4 (2.67%)	0.684	2 (1.69%)	1 (1.69%)	1 (1.69%)	1.000
Intra-abdominal infection	15 (6.12%)	5 (5.26%)	10 (6.67%)	0.655	5 (4.24%)	3 (5.08%)	2 (3.39%)	1.000
Intra-abdominal Bleeding	8 (3.27%)	3 (3.16%)	5 (3.33%)	1.000	3 (2.54%)	2 (3.39%)	1 (1.69%)	1.000
Medical complications	49 (20.00%)	13 (13.68%)	36 (24.00%)	0.049*	17 (14.41%)	5 (8.47%)	12 (20.34%)	0.066
Pleural and peritoneal effusion	13 (5.31%)	4 (4.21%)	9 (6.00%)	0.543	6 (5.08%)	2 (3.39%)	4 (6.78%)	0.675
Pulmonary complications	14 (5.71%)	4 (4.21%)	10 (6.67%)	0.420	5 (4.24%)	1 (1.69%)	4 (6.78%)	0.361
Venous thrombosis	8 (3.27%)	2 (2.11%)	6 (4.00%)	0.657	4 (3.39%)	2 (3.39%)	2 (3.39%)	1.000
Hypoalbuminemia	3 (1.22%)	0 (0.00%)	3 (2.00%)	0.429	2 (1.69%)	0 (0.00%)	2 (3.39%)	0.476
Multiple complications	11 (4.49%)	3 (3.16%)	8 (5.33%)	0.423	7 (5.93%)	3 (5.08%)	4 (6.78%)	1.000

Values are shown as n (%) unless otherwise indicated.

a Postoperative complications in this study were defined as any adverse event corresponding to Clavien-Dindo classification grade, occurring within 30 days after surgery. If a patient had more than one type of complication, the complication with the highest grade was used for the analysis.

*P<0.05, statistically significant.

### Risk factors for postoperative complications

A risk analysis of the overall postoperative complications was performed to investigate the risk factors for postoperative complications. Univariate and multivariate analyses of the factors associated with overall postoperative complications before and after PSM are summarized in **Table 4**. Univariate analysis revealed that open surgery, age≥65 years, and non-BI reconstruction were significant risk factors. Moreover, laparoscopic surgery (odds ratio [OR] 0.263; 95% CI 0.116-0.597; P = 0.001) and B-I reconstruction (OR 0.502; 95% CI 0.278-0.908; P = 0.023) were identified as independent protective factors in the multivariable analysis before PSM. After PSM, it was also found that laparoscopic surgery (OR 0.099; 95% CI 0.022-0.454; P = 0.003) and B-I reconstructions (OR 0.366; 95% CI 0.161-0.833; P = 0.017) independently associated with the less rate of postoperative complications.

**Table 4:** Univariate and multivariate logistic analysis of factors associated with total postoperative complications.

Factors	Un-matched				Matched			
	Univariate analysis		Multivariate analysis		Univariate analysis		Multivariate analysis	
	OR (95% CI)	P	OR (95% CI)	P	OR (95% CI)	P	OR (95% CI)	P
Gender		0.824				0.556		
Female	1				1			
Male	1.073 (0.577-1.993)				1.310 (0.534-3.210)			
Age (y)		0.043*		0.270		0.278		
≤65	1		1		1			
>65	1.736 (1.017-2.964)		1.374 (0.782-2.414)		1.537 (0.707-3.338)			
NRS 2002 score		0.235				0.322		
1-2	1				1			
3-4	1.316 (0.722-2.397)				1.440 (0.603-3.440)			
5-6	2.250 (0.823-6.152)				2.770 (0.667-10.923)			
ASA grade		0.351				0.104		
1-2	1				1			
3-4	1.352 (0.717-2.550)				2.033 (0.865-4.780)			
Hypertension		0.927				0.520		
No	1				1			
Yes	0.975 (0.567-1.676)				0.768 (0.344-1.716)			
Diabetes mellitus		0.289				0.148		
No	1				1			
Yes	1.412 (0.746-2.671)				1.938 (0.790-4.754)			
Previous abdominal surgery		0.655				0.397		
No	1				1			
Yes	1.195 (0.547-2.611)				1.553 (0.561-4.296)			
Tumor location		0.256				0.173		
Antrum	1				1			
Body	0.840 (0.419-1.685)				0.067 (0.002-2.063)			
Cardia	0.849 (0.902-3.793)				0.661 (0.040-10.885)			
Total	0.616 (0.120-3.161)				1.000 (0.020-50.397)			
Differentiated degree		0.692				0.504		
Differentiated	1				1			
Undifferentiated	0.715 (0.282-1,810)				0.464 (0.120-1.795)			
Signet ring carcinoma	1.157 (0.552-2.423)				1.093 (0.422-2.829)			
Pathological type		0.443				0.547		
Ulcerative type	1				1			
Non-ulcerative type	1.383 (0.604-3.116)				1.529 (0.383-6.104)			
TNM stage						0.621		
I	1	0.693			1			
II	1.340 (0.677-2.654)				0.947 (0.352-2.549)			
III	1.196 (0.647-2.210)				0.667 (0.272-1.634)			
Laparoscopic gastrectomy						0.004*		
No	1	<0.001*	1	0.001*	1		1	0.003*
Yes	0.216 (0.097-0.481)		0.263 (0.116-0.597)		0.108 (0.024-0.486)		0.099 (0.022-0.454)	
B-I		0.002*		0.023*		0.022*		0.017*
No	1		1		1		1	
Yes	0.407 (0.230-0.719)		0.502 (0.278-0.908)		0.404 (0.186-0.880)		0.366 (0.161-0.833)	

BMI, body mass index; ASA, American Society of Anesthesiologists; NRS 2002, nutritional risk screening 2002, TNM, tumor-node-metastasis.

*Statistically significant (P < 0.05).

### Risk factors for OS

**Figure 3:**
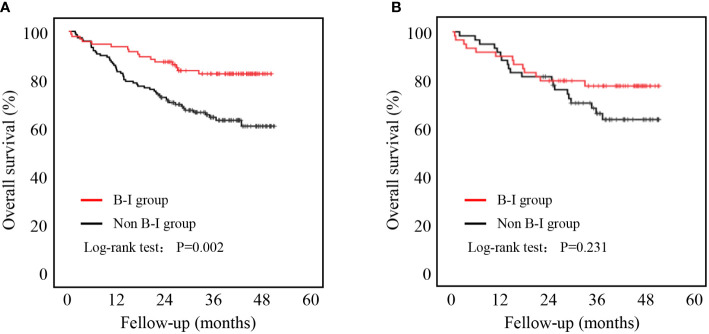
Five-year overall survival curve calculated using the Kaplan-Meier method comparing B-I and non-B-1. **(A)** Before PSM; **(B)** After PSM.

As shown in **Figure 3**, patients in the B-I group had better outcomes than those in the non-B-I group before PSM (P=0.002). Further evaluation of the potential factors influencing OS revealed that it was affected by age (HR 2.435, 95% CI 1.435-4.132, P=0.001), NRS score (NRS 3-4: HR 1.100, 95% CI 0.634-1.907; NRS 5-6: HR 3.886, 95% CI 1.978-7.635, P<0.001), tumor location (Body: HR 0.946, 95% CI 0.483-1.851; Cardia: HR 1.652, 95% CI 0.895-3.050; Total: HR 4.850, 95% CI 2.042-11.521, P=0.002), tumor TNM stage (TNM stage II: HR 2.447, 95% CI 1.026-5.834; TNM stage III: HR 6.332, 95% CI 2.986-13.426, P<0.001), laparoscopic surgery (HR 0.173, 95% CI 0.063-0.474, P=0.001) and B-I reconstructions (HR 0.419, 95% CI 0.239-0.733, P=0.002) on univariate analysis. Among them, only tumor TNM stage (TNM stage II: HR 1.897, 95% CI 0.777-4.629; TNM stage III: HR 4.544, 95% CI 2.077-9.944, P<0.001) and laparoscopic surgery (HR 0.270, 95% CI 0.095-0.765, P=0.014) were independently associated with OS (**Table 5**).

**Table 5:** Univariate and multivariate COX regression analysis of factors associated with overall survival.

Factors	Un-matched				Matched			
	Univariate analysis		Multivariate analysis		Univariate analysis		Multivariate analysis	
	HR (95% CI)	P	HR (95% CI)	P	HR (95% CI)	P	HR (95% CI)	P
Gender		0.428				0.520		
Female	1				1			
Male	1.268 (0.705-2.280)				1.315 (0.571-3.031)			
Age (y)		0.001*		0.054		0.102		
≤65	1		1		1			
>65	2.435 (1.435-4.132)		1.729 (0.991-3.015)		1.858 (0.884-3.905)			
NRS 2002 score		<0.001*		0.296		0.260		
1-2	1		1		1			
3-4	1.704 (0.997-2.912)		1.100 (0.634-1.907)		1.593 (0.741-3.428)			
5-6	3.886 (1.978-7.635)		1.773 (0.861-3.652)		2.146 (0.730-6.312)			
ASA grade		0.344				0.474		
1-2	1				1			
3-4	1.303 (0.753-2.252)				1.312 (0.624-2.757)			
Hypertension		0.696				0.800		
No	1				1			
Yes	1.102 (0.677-1.794)				1.096 (0.539-2.228)			
Diabetes mellitus		0.694				0.979		
No	1				1			
Yes	1.122 (0.633-1.988)				1.011 (0.439-2.330)			
Previous abdominal surgery		0.521				0.883		
No	1				1			
Yes	1.245 (0.637-2.434)				0.931 (0.359-2.411)			
Tumor location		0.002*		0.144		0.012*		0.012*
Antrum	1		1		–		–	
Body	0.946 (0.483-1.851)		1.223 (0.509-2.055)		–		–	
Cardia	1.652 (0.895-3.050)		1.238 (0.639-2.399)		–		–	
Total	4.850 (2.042-11.521)		2.9245 (1.174-7.283)		–		–	
Differentiated degree		0.534				0.895		
Differentiated	1				1			
Undifferentiated	1.350 (0.641-2.843)				0.802 (0.240-2.675)			
Signet ring carcinoma	0.772 (0.367-1.623)				1.110 (0.476-2.588)			
Pathological type		0.627				0.538		
Non-ulcerative type	1				1			
Ulcerative type	0.840 (0.417-1.694)				0.638 (0.153-2.667)			
TNM stage		<0.001*		<0.001*		0.001*		0.002*
I	1		1		1		1	
II	2.447 (1.026-5.834)		1.897 (0.777-4.629)		1.392 (0.374-5.186)		1.333 (0.358-4.966)	
III	6.332 (2.986-13.426)		4.544 (2.077-9.944)		4.939 (1.711-14.256)		4.736 (1.636-13.705)	
Laparoscopic gastrectomy		0.001*		0.014*		0.053		
No	1		1		1			
Yes	0.173 (0.063-0.474)		0.270 (0.095-0.765)		0.309 (0.094-1.014)			
B-I		0.002*		0.590		0.216		
No	1		1		1			
Yes	0.419 (0.239-0.733)		0.840 (0.446-1.582)		0.644 (0.320-1.294)			

BMI, body mass index; ASA, American Society of Anesthesiologists; NRS 2002, nutritional risk screening 2002, TNM, tumor-node-metastasis.

*Statistically significant (P < 0.05).

We further compared the OS rates between the two groups after PSM. There was no significant difference in OS between the two groups (P = 0.231, **Figure 3**).

## Discussion

BMI, conveniently calculated as the patient’s weight divided by the square of height, has been broadly used as an indicator of obesity (18, 20). However, BMI cannot distinguish the fat distribution in the abdominal cavity (13). Recently, studies proposed that visceral fat was a better tool for predicting surgical outcomes (21, 22). Considering the priority of VO over BMI in estimating visceral fat, we used CT-based VFA for determining VO. In this way, we focused on patients with VO to compare postoperative complications and OS upon different post-gastrectomy reconstruction methods using PSM. We found that the overall postoperative complications in the B-I group were significantly lower than those in the non-B-I group, while no differences in OS were found between the different reconstruction methods. In addition, BI reconstruction was found to be a strong independent protective factor for postoperative complications.

It is known that B-I reconstruction is most commonly performed due to its technical simplicity and intervention in a single anastomotic site, as well as the preservation of the physiological path (23, 24). In contrast, B-II and R-Y reconstructions are more applicable because they are simpler techniques after distal gastrectomy. Although these techniques for digestive tract reconstructions have already been compared in their short-term complications and long-term prognoses (7, 25), no consistent results have been reached so far, and the preferred reconstruction method remains controversial. In addition, some aspects, such as which anastomosis is more appropriate for patients with VO and GC have not been explored, and this is the first study evaluating this factor.

Here, the B-I group had a decreased incidence of overall postoperative complications. After further performing PSM to balance the deviation of tumor characteristics and the patients’ general condition, we also found that B-I reconstruction was still an independent protective factor for postoperative complications, consistent with a previous multi-institutional study (6). In addition, the difference was mainly concentrated in mild postoperative complications, as most patients with complications were treated conservatively, and severe complications were unusual among the patients. However, although we found that nearly all complications had a lower incidence in the B-I group, no statistical difference was found, possibly due to the small sample size after matching. Additionally, considering the small sample size, we took patients underwent total gastrectomy and distal gastrectomy together rather than analyzed separately. However, since the patients underwent total gastrectomy were relatively few, our results are mainly representative of distal gastrectomy. All in all, a large-scale study is needed to characterize this aspect further.

Another concern in selecting reconstruction methods is the long-term prognosis, as it has been shown (26, 27) that a delay in chemotherapy due to postoperative complications adversely affects the OS of patients with GC. Previous studies showed a non-significant difference in the 5-year OS rates between the B-I, B-II, and R-Y reconstruction groups (25). Similarly, we found that B-I reconstruction yielded oncologic outcomes comparable to those of non-B-I reconstruction. However, our results demonstrated that OS was significantly longer in patients who underwent B-I reconstruction before PSM. This may be attributed to the TNM stage, which greatly affects the prognosis and differs between the groups, as patients undergoing B-I reconstruction showed a lower TNM stage. As reported in a previous study (25), although the range of surgical dissociation varied, gastrectomy and lymph node dissection for GC were so standardized that the digestive tract reconstruction method did not affect the number of retrieved lymph nodes or the prognosis. Therefore, it is more acceptable that B-I reconstruction was not an independent risk factor for OS.

To the best of our knowledge, this is the first study to evaluate the short- and long-term outcomes of different reconstruction methods in GC patients with VO using PSM analysis. However, this study had several limitations. First, this was not a randomized controlled trial, and inherent selection biases exist that can be adjusted but not completely eliminated using PSM. Additionally, this was not a multicenter study, and our results may not be directly applicable to other populations. Finally, although the patients’ baseline data was well balanced after PSM, the small sample size may greatly limit our conclusions as patients underwent laparoscopic or open surgery, total gastrectomy or distal gastrectomy should be discussed separately as surgical method may also greatly affected the postoperative complications, thus a large-scale study is necessary.

## Conclusions

We compared the incidence of postoperative complications between different reconstruction methods among GC patients with VO and found that B-I reconstruction can reduce the incidence of postoperative complications, thus promoting postoperative recovery. However, no significant differences in OS were found among the three reconstruction methods. Last, because of its implementation facility compared to the other two approaches, B-I reconstruction may be considered a better choice for patients with GC with VO.

## Data availability statement

The original contributions presented in the study are included in the article/supplementary material. Further inquiries can be directed to the corresponding authors.

## Ethics statement

All procedures involving human participants performed in the study followed the Helsinki declaration and was approved by the Ethics Committee of the Second Affiliated Hospital of Wenzhou Medical University in China. All individual participants included in the study signed informed consent to participate in the study.

## Author contributions

XYX, XS and SW were the study designs. CM, HY and JC collected and analyzed data. CM, MX and XFX wrote the manuscript and interpreted it. WC, JW, JZ and XC revised the paper. The authors read and approved the final manuscript. All authors contributed to the article and approved the submitted version.
